# Appalachian Caregiver Perspectives on Childhood Gun Safety in the Home

**DOI:** 10.13023/jah.0301.04

**Published:** 2021-01-24

**Authors:** Dannell Boatman

**Affiliations:** West Virginia University Cancer Institute

**Keywords:** Appalachia, gun safety, childhood injury, injury prevention

## Abstract

**Background:**

Childhood gun injuries pose a critical public health challenge. For children, unintentional gun injury deaths primarily occur in the home where parents or other adult guardians, referred to as caregivers hereafter, are responsible for safety. While the American Academic of Pediatrics recommends not having guns in areas where children live and play, firearms are often viewed as normative and fill an important role in many homes. This is particularly true in more rural areas, such as Appalachia, where there is a high density of gun ownership. Additional research is needed to understand rural caregivers’ current gun safety practices in the home.

**Purpose:**

The purpose of this study was to gain an understanding of Appalachian caregivers’ gun safety practices, perspectives, and attitudes to assist public health professionals develop more effective interventions and targeted messaging.

**Methods:**

Ten Appalachian caregivers were interviewed for a qualitative, phenomenologic study designed to elicit an in-depth understanding of firearm safety strategies in the home. An inductive analytic approach to coding and analysis was used to identify main themes and ideas.

**Results:**

Current attitudes, practices, and perspectives focused on the primary childhood injury prevention strategies of education, environmental change, and supervision. Findings matched and expanded upon previous literature in the field.

**Implications:**

Cross-cutting themes were identified that have practical implications for the development of public health interventions and messaging for this at-risk population.

## BACKGROUND

Each year in the United States, childhood gun injuries lead to approximately 1300 deaths and 5800 injuries that require medical treatment.^1^ Most unintentional firearm injury deaths in childhood occur within the home.^2^ At least one gun is present in approximately 29% of homes with children under the age of 18 years,^3^,^4^ even though the American Academy of Pediatrics recommends that guns should not be in areas where children live or play.^5^ Despite data to suggest that guns in the home increase the risk of injury, there is a belief that having immediate access to firearms makes homes safer.^4^ See the Additional Files for an infographic of this article.

Gun ownership is deeply rooted in rural culture and is seen as part of an individual’s identity.^5^,^6^ Compared to residents in urban areas who often view guns in a negative light, residents in rural areas view firearms more positively, as parts of their tradition and heritage.^7^ Rural communities have a higher level of gun ownership.^4^,^5^ Children living in one of five states (West Virginia, Arkansas, Mississippi, Alabama, Louisiana) with the highest numbers of firearm availability are 16 times more likely to die of an unintentional gun injury compared with the five states with the lowest gun availability.^8^,^9^ This suggests that accessibility and availability of firearms increases gun injury risk, making rural areas particularly susceptible.^8^,^10^ An understanding of the perspectives of individuals immersed in distinct cultures, like Appalachia, is needed to develop more effective gun injury prevention strategies.^11^

### Injury Prevention Strategies in the Home

Parents or other adult guardians (referred to as caregivers hereafter) use three primary strategies to prevent childhood injuries: supervision, environmental change, and education.^12^ To assess gun safety in the home, there needs to be an understanding of caregivers’ current practices related to these primary injury prevention strategies.

Supervision has been cited as the most effective primary prevention strategy to reduce injury in children.^13^,^14^ Levels of supervision vary according to how a caregiver perceives the danger of a situation and their child’s characteristics.^13^,^15^ There is some limited evidence illustrating a potential disconnect between how caregivers perceive their child’s interest in guns compared with how the child reports interacting with firearms.^16^ There are no identified studies assessing Appalachian caregiver supervision practices related to gun safety.

Safe gun storage has been found to be an essential environmental change to reduce childhood firearm injuries.^17^ While caregivers acknowledge that gun storage is critical for safety, only 3 in 10 adults with children in the household report storing all guns locked and unloaded.^4^ Further, caregivers often believe that children will be unable to find their guns in the home.^18^ With that said, a clear understanding of how caregivers store their guns is unknown.^19^ There are no identified studies on the gun storage practices of Appalachian caregivers.

As children age, caregivers begin to rely on teaching safety behaviors more than supervision or modifying the environment.^12^,^20^ This transition to education as the primary safety strategy leads to an increased risk of injury.^12^ At this point, caregivers begin to believe that their child can understand safe behaviors there is little reason to adhere to all safety recommendations.^12^ Taken collectively with a potential disconnect between caregivers and children on levels of interest and behavioral tendencies toward firearms in the house,^16^ this could lead to potentially dangerous situations. Further, little is known about the frequency, format, or content of gun safety conversations between caregivers and children.^19^ There are no identified studies on Appalachian caregiver gun safety conversations or educational strategies.

## PURPOSE

The purpose of this study was to gain an understanding of Appalachian caregivers’ gun safety practices, perspectives, and attitudes. Through this increased understanding, public health professionals could develop more effective safety interventions and targeted messaging.

## METHODS

A phenomenologic approach was selected as the methodology for this qualitative study. As there is little existing literature exploring caregivers’ attitudes, perspectives, and practices regarding firearm safety in the home, and no identified studies focusing solely on the Appalachian population, qualitative methods were needed to identify themes for future research. This study was exempted by A.T. Still University Institutional Review Board under Section 45CFR46.104(d)(2)(i) in February 2020.

A sample unit for this study was an adult caregiver (aged 18 years or older) with at least one child (biological or adopted) between the ages of 2 and 10 years living in the home at least part-time. Participants lived in Appalachia, primarily West Virginia, and owned at least two personal firearms that were stored within the home. This study was highly targeted, so a purposeful sampling strategy was chosen. Participants were recruited from parenting groups, primarily associated with preschool-aged child programming, housed within a community center located in northern West Virginia. This community center was selected due to existing relationships with parenting group leaders which would support the recruitment process.

Semi-structured interviews were used to collect data for analysis. Instrumentation included a participant demographics form and an Interview Guide (see Additional Files), which was validated through a pilot study. In-person interviews were conducted at the community center, each lasting approximately 30–45 minutes. Recruitment for this study began in February 2020 and was active until saturation was achieved in early April 2020. Saturation for this project was defined as acquiring no new themes or codes through participant interviews.

Four onsite interviews at the community center were staggered to reach different parenting groups. After the study was introduced, data were collected from a private room for the length of the session, approximately 3 hours. Potential participants were encouraged to go to this room to be screened for eligibility, receive informed consent, and complete the interview. Each participant received a $10 Walmart gift card at the conclusion of the interview.

After an interview was completed, a study identification number was assigned, and all personal identifiers were removed from study documentation. Audio recordings of the interviews were transcribed by QSR International NVivo Transcription Services. After the draft transcripts were returned, audio recordings were reviewed while checking the transcript for accuracy. Each participant’s case in NVivo 12, the platform that supported the data management and analysis process, was linked to all relevant study documentation to ensure its completeness.

After the data were cleaned, the analysis process began by coding interview transcripts. Coding occurred within NVivo 12. Main categories were developed in a codebook and tied to study research questions, which followed an inductive analytic approach. As new topics and themes were identified in each of the main categories, sub-nodes were added clarify thoughts for analysis and synthesis.

Credibility for the coding process was established by comparing transcripts coded for the study with those coded by a colleague. After the coding process was complete, member checking was used with several participants to validate findings and further establish accuracy and credibility for the study. Bracketing, specifically journaling, was used to promote objectivity throughout the study.

## RESULTS

Ten participants were included in the study (Table 1). Findings were organized into four key areas which aligned with childhood injury prevention strategies: gun safety education, gun storage practices, child interest in guns, and caregiver attitudes. Three cross-cutting themes and their potential affect on intervention development were identified as the key areas were synthesized collectively.

### Gun Safety Education

All study participants suggested that teaching children about guns was a critical safety strategy. Most participants indicated that teaching gun safety was more important when children grew up with guns in the home. All participants suggested teaching children about firearms early, although participants differed on the specific age to begin these safety conversations.

#### Developmentally linked educational approaches

Most participants viewed educational approaches to gun safety as linked to a child’s developmental capabilities. A child’s characteristics and ability to comprehend information was viewed as an important consideration to caregivers as they decided when to begin safety conversations. One participant described a gun safety conversation with her young child:

[The first conversation focused on] how serious they [guns] are, they’re not toys, and even the toy ones, we shouldn’t be shooting people in the face. I’d rather not even shoot at people. Although water guns and stuff, you know, gosh, it’s hard. But I do remember us, without trying to traumatize him, explaining that somebody can die, that it’s not a game or a toy, and if you ever find a gun to go tell somebody, to not touch it and you go tell somebody.

As children aged, gun safety conversations typically became more informal and linked with the child’s introduction to hunting or other firearm-related recreational activities important to the family. One participant said: “You go from talking about this could really hurt you to by the time you’re 12 years old, you can take your hunter safety course to get your hunting license.”

#### Messaging

Messaging for safety-related conversations between children and participants focused on two themes: avoidance and proper use (Table 2). If children were not deemed to be developmentally capable of understanding more detailed information about firearms, caregivers focused on avoiding guns, telling a caregiver if they saw one, and recognition of fake versus real firearms. As children were perceived by caregivers as maturing, the conversations focused more on proper use of firearms, safety strategies when using guns, and how to react when they see one within the home. One participant described shifting messaging:

The 6-year-old it’s more like “don’t do this.” The 10-year-old is kind of like, “if you see this, this is what we do,” and then the 14-year-old, it’s like, “if you go into a house and you see dad’s gun laying there, what would you do?” I think it is kind of like in steps.

#### Teaching strategies

Caregivers suggested that active, hands-on approaches to teaching children about firearms were best. A typical activity described by participants included showing younger children real and toy guns so that they could recognize the difference. For older children, caregivers used real guns and taught proper use.

#### Teachers

With guns in the house, most participants felt the responsibility of teaching gun safety rested with them. While some participants felt that no one else should teach their children about guns, others felt that experienced friends and family members were reliable, trusted sources of information for their children. Outside of immediate relationships, police officers and gun course instructors were identified by participants as individuals they felt could help teach children about gun safety.

### Gun Storage Practices

**Figure 1:**
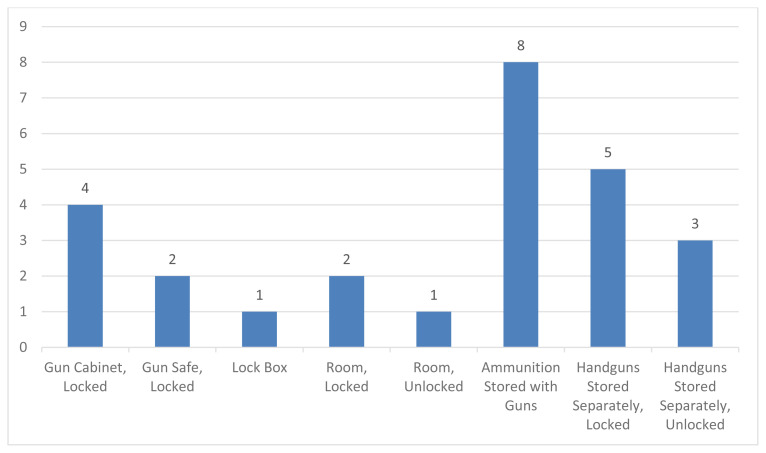
Participant Gun Storage Strategies (categories not mutually exclusive)

All participants indicated that gun storage was a critical safety strategy in the home. In addition, all participants used some form of storage to keep their children away from guns (Figure 1). Most participants were confident that their gun storage strategies would keep their children safe. Many felt that the guns were securely locked, in spaces where children are not allowed to go, and/or children would not be able to find or reach them.

#### Home protection

Handguns, viewed primarily as a means of home protection, were frequently stored differently or separately than other gun types that were viewed as more recreational. Handguns were typically in a separate lock box close to the bed, found unlocked in the caregiver’s bedroom, or another easier to access location. In some situations, the handgun remained loaded or the ammunition was nearby for quick access. A participant articulated this belief, “If it’s not loaded and ready to go, it’s pretty much worthless because that one second could be the difference between life and death.”

#### Learning storage strategies

Six of the 10 participants grew up around guns and reported learning storage strategies from their family members. “My grandfather, he told me just to always keep them [guns] locked up. It was passed through the family.” Friends with an interest in firearms were also seen as valued sources of knowledge on gun storage strategies. Participants with less firearm experience relied on their partners or spouses who were perceived to have more knowledge on the topic.

#### Changing storage strategies

Most participants indicated they did not need to change storage strategies as their children aged, expressing comfort with the methods they had in place. Several stressed a heightened vigilance in making sure guns were secured and in locked areas once children became mobile.

#### Challenges to gun storage

Most participants indicated that there were few challenges or barriers to safely securing guns in the home. Of those who indicated challenges, the cost of gun cabinets and safes were problematic. Other forms of safe gun storage, such as trigger locks, were viewed as repetitive and unnecessary burdens. One participant spoke to gun safe expense, “Buying the safes are a little expensive, but you’ve got to weigh the pros and cons of how safe you want your family to be. You don’t want the four-year-old accidentally getting a hold of a loaded gun.”

### Child Interest in Guns

Most participants felt that their children had little interest in the firearms located within the home. Several participants felt that their children did not know guns were in the home, so their interest had not yet been piqued. “No, I don’t think he knows they’re in the house and he doesn’t have any toys that have guns. I think at this point he wouldn’t know what it is if he were to see it.” Other participants had conversations with their children but did not observe an increased interest beyond participation in family gun-related activities.

#### Supervision

Most participants suggested that other safety strategies, such as storage and conversations, mitigated the need for constant supervision when children were in spaces where guns were located. Conversations about guns and gun safety were seen as a way to build trust with children around guns. Participants also felt that strong storage strategies meant that they could reduce supervision.

### Caregiver Attitudes Toward Guns

Most participants viewed guns as an important part of their culture and lifestyle. More than half of the participants were raised around guns and had family members and friends who engaged in similar activities, such as hunting.

#### Child perception of guns

Most participants expressed the desire for their children not to fear guns in the home or view them as a threat, but to understand their uses and interact with them safely. Most participants expressed a desire to involve children in their gun-related activities when they were developmentally ready. One participant noted:

I don’t think it [gun] has to be something you’re scared of if you understand how to use it safely and how it can be harmful if you’re not going to use it safely. Not trying to tell him [child] that to scare them, but to understand the whole power of that weapon.

### Cross-Cutting Themes

Cross-cutting themes were identified through the data analysis process (Table 3).

#### Gun lifestyle/culture

All participants in this study, even those not raised with guns in the home, expressed a personal positive view of guns and being a part of the gun lifestyle and culture. They expressed a strong desire to include their children in this lifestyle with a positive view of guns and an appreciation of their uses. This suggests that any approaches to change gun safety behaviors with Appalachian caregivers should come from a place of knowledge and appreciation of this culture. Most participants viewed friends, family, police officers, and gun course instructors as reliable sources of information on gun safety. These stakeholders should be involved in the intervention and messaging development to lend credibility to the process.

#### Developmentally linked gun safety strategies

Most participants indicated the need to tailor their messaging, level of supervision, and storage strategies to the age and characteristics of each child. They modified safety approaches to reflect perceived changes in child comprehension and development. Interventions to improve or change safety strategies with Appalachian caregivers should reflect developmentally appropriate approaches, which was important to the study population.

#### Home protection/security

While participant recreational and hunting firearms were often stored safely, handguns were often viewed as an exception. These guns were seen as essential to home security so many participants felt that adding layers of storage made their homes less safe. Public health professionals designing gun safety interventions for this population should take this viewpoint into consideration and suggest ways to address firearm safety concerns while recognizing this strongly held belief.

## IMPLICATIONS

To develop effective childhood gun safety interventions, public health professionals should understand the practices, attitudes, and perspectives of at-risk populations, including rural Appalachians. Through this phenomenologic study, themes important for this population were identified. By understanding these current practices, public health professionals can tailor interventions more effectively. In addition, three cross-cutting themes and their potential effect on childhood gun safety intervention and messaging development were identified.

As this was a phenomenologic study, a small number of participants from one location were used for in depth study. Because of this, the results are not generalizable outside this group. In addition, the demographics of the participants were not representative of Appalachia overall. As this study relied on voluntary participation, self-selection bias was a potential limitation as well. Researcher bias was reduced through bracketing and member checking, though still a potential limitation. Future research should explore the identified themes within a broader swath of the Appalachian population. In addition, the need to explore these themes with caregivers that have older children should also be considered. Finally, the need to expand study beyond unintentional injury to self-harm is warranted.

**SUMMARY BOX**

**What is already known about this topic?** Rural states have greater accessibility to and availability of guns, making children in these areas more susceptible to unintentional injuries.

**What is added by this report?** There have been no identified studies on Appalachian caregiver practices, attitudes, and perspectives toward gun safety in the home. This qualitative study provides key themes that can guide future research and public health intervention and messaging development for this at-risk population.

**What are the implications for future research?** Unintentional gun injuries cause various negative consequences, including physical, mental, and economic challenges that expand past the shooting victim. An understanding of current caregiver safety practices in the home is needed to develop more effective prevention strategies and policies for an at-risk population traditionally hesitant to discuss gun-related issues.

## Supplementary Information





## Figures and Tables

**Table 1:** Participant Demographics

Study ID	Gender	Age Range	Race/Ethnicity	Education	Marital Status	Children	Guns Owned
P01	M	26–49	White	High School	Married	2	11–25
P02	F	26–49	White	Vocational School	Married	2	1–10
P03	F	26–49	White	Bachelor’s Degree	Married	2	1–10
P04	F	26–49	White	Some College	Divorced	2	1–10
P05	F	26–49	White	High School	Married	2	1–10
P06	F	26–49	White	Bachelor’s Degree	Married	3	1–10
P07	M	18–25	Black	High School	Never Married	1	1–10
P08	F	26–49	White	Advanced Degree	Married	2	1–10
P09	F	18–25	White	Vocational School	Never Married	1	1–10
P10	M	26–49	White	Bachelor’s Degree	Married	2	1–10

**Table 2:** Developmentally Influenced Educational Messaging and Teaching Methods

Developmentally Influenced Strategy	Messaging and Teaching Methods
Avoidance Younger children deemed not developmentally ready for in-depth information on gun safety	Formal conversations Hands-on teaching methods ▪ Mimicking safe behavior with toy guns (i.e., not pointing toy guns at people) ▪ Showing differences between real and toy guns ‘Avoidance’ messaging ▪ Emphasizing guns are not toys ▪ Explaining uses (i.e., hunting for food) ▪ Emphasizing the potential to be seriously hurt or harm to others ▪ Instructing child to avoid guns ▪ Encouraging child to tell adult if they see a gun in the home
Proper Use Older children deemed developmentally ready for in-depth information on gun safety	Informal conversations during gun-related activities Hands-on teaching methods ▪ Using real guns to provide children with hands-on practice ‘Proper use’ messaging ▪ Conversations on safe gun use (i.e., safety on, not pointing at people, finger off trigger) ▪ Sharper messages on consequences of unsafe use ▪ Trusting children to know how to react and respond to guns they see in the home

**Table 3:** Cross-Cutting Themes and Potential Effect on Gun Safety Interventions

Theme	Potential Impact on Interventions
Gun Lifestyle/Culture Firearms are viewed positively in the home and children are expected to become an active participant in the family’s gun culture/lifestyle	Appreciation of this culture/lifestyle is needed when designing interventions Knowledgeable stakeholders within this culture need to be involved in intervention development to establish credibility
Developmentally Linked Gun Safety Strategies Caregivers viewed storage, educational, and supervision strategies as linked closely with individual child characteristics and development	Interventions need to be described on a continuum based on child characteristics and development to provide practical advice to caregivers
Home Protection/Security Pistols and/or handguns are often stored less safely than other guns to ensure easy access to protect the home	Interventions need to reflect this belief to be relevant and relatable to this population
