# Supplementary material for: Appalachian Caregiver Perspectives on Childhood Gun Safety in the Home
Source: J Appalach Health. 2021 Jan 24;3(1):29–42. doi: 10.13023/jah.0301.04 (PMC9138731; doi:10.13023/jah.0301.04)
Supplement: Supplementary file 1 [file Infographicfor3.1.4Boatman.pdf]

Childhood Gun Safety in the Home: Perspectives of Appalachian Parents

Study Overview

Ten parents of children under the age of 10 in northern West Virginia were recruited for semi-structured interviews to learn about their gun safety practices in the home. Through this increased understanding of attitudes, perceptions, and practices, public health professionals can develop more effective gun safety messages and interventions to reach this population.

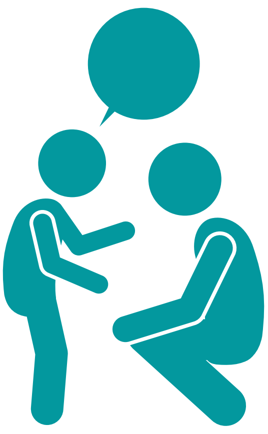

Teaching Gun Safety to Children

Study participants felt that it was important to teach their children about gun safety early, but differed on the specific age to begin (ages 2 through 8 were suggested). Parents focused on avoidance messaging when children were younger and transitioned to a focus on proper use when the child was viewed as developmentally ready to engage in firearm-related activities. The age when this transition occurred varied due to parental perception of individual child characteristics.

Storing Guns in the Home

Most study participants felt that their guns were locked or hidden away and that their children could not access them. Guns were usually not loaded but ammunition was often kept in the same area. Participants did not adhere to all American Academy of Pediatrics recommendations, instead relying on storage approaches learned from family or friends. Participants often stored handguns, viewed as critical to home security, without a lock or loaded.

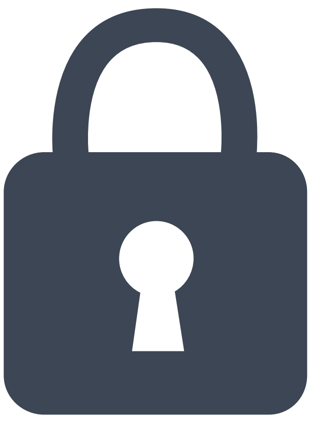

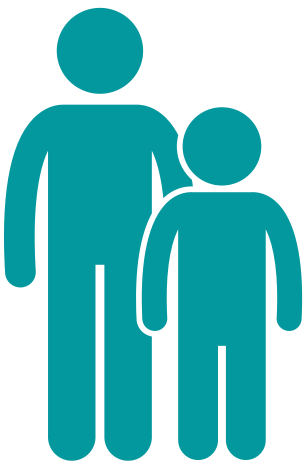

Child Interest in Guns

Study participants felt that they did not need to focus heavily on child supervision unless guns were out of storage for activities or cleaning. Most participants indicated that their children did not express interest in guns. Children who were already participating in firearm-related activities were seen to be more interested in guns than those viewed as too young to participate.

Parental Attitudes

Study participants viewed guns as an important part of their culture and lifestyle. They were excited to involve their children in firearm-related activities when they were deemed capable of handling the responsibility. Participants wanted their children not to grow up in fear of guns but to understand the risks and how to handle them properly to mitigate them.

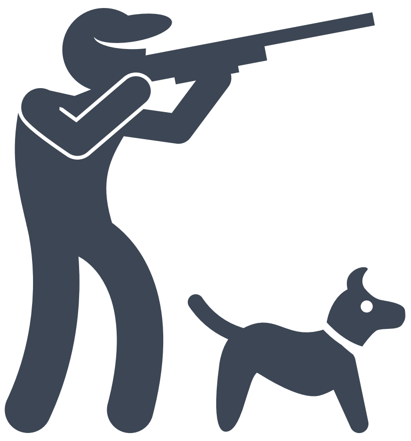

Practice Implications

Study themes were identified that may have implications for intervention development.

Gun Lifestyle/Culture

Firearms were viewed positively in these homes and children were expected to become active participants in this lifestyle. **Why does this matter?** An appreciation of this culture is needed when designing interventions or developing messaging. It is critical to involve knowledgeable stakeholders who understand this lifestyle during intervention development.

Developmentally Linked Gun Safety Strategies

Parents linked storage, education, and supervision strategies closely with individual child characteristics and development. **Why does this matter?** Developing interventions based on a developmental continuum may make sense to this population and encourage adoption of strategies.

Home Protection

Handguns are often stored less safely than other guns to ensure easy access for home protection. **Why does this matter?** Interventions need to recognize and address this belief to be relevant and relatable to this population.
