# Supplementary material for: Appalachian Caregiver Perspectives on Childhood Gun Safety in the Home
Source: J Appalach Health. 2021 Jan 24;3(1):29–42. doi: 10.13023/jah.0301.04 (PMC9138731; doi:10.13023/jah.0301.04)
Supplement: Supplementary file 2 [file InterviewGuide-for3.1.4Boatman.pdf]

Interview Guide

1. Introduction

    - Thank you for agreeing to this interview. My name is INTERVIEWER, and I am a doctoral student studying health education.
    - The purpose of this interview is to understand the different strategies caregivers use to keep their children safe around guns in the home.
    - There are no right or wrong answers to these questions. I am just trying to understand what works for you.
    - If you do not feel comfortable answering a question, tell me, and we can skip it.
    - Whatever you share with me is confidential. Your name and all identifiers in your responses will be removed from this research.
    - If it is ok, I would like to record this interview, so I do not miss any of your answers. I would also like to take notes as you talk.
    - Here is an informed consent form describing your voluntary participation in this study. Please review it and sign it if you agree to participate.
    - Thank you for signing. Before we get started with the interview, could you please take a minute or two to fill out this information sheet? This will help me understand some basic demographic information for study participants. Again, this information is confidential and will not be linked to your name.

2. Please describe your personal use of firearms.
    - Who uses the firearms in your home?

3. Could you tell me about your introduction to firearms?
    - What age do you remember seeing/holding/shooting a firearm for the first time?
    - Could you tell me how you first learned about gun safety?
        - Describe some of the safety messages you were taught.
        - Who were the individuals that helped you learn about gun safety?

4. Please describe how you store firearms in your home.
    - Where do you store your guns?
    - When do you store your guns?
    - Do you feel like these gun storage strategies are important for your child's safety?
        - Why or why not?
    - Where did you learn these gun storage strategies?
    - Do you feel like you could improve your current gun storage strategies?
        - If so, what would you do?
    - Please describe any barriers or challenges to gun storage in your home.

- Have you made any changes to your gun storage practices when you had a child?
    - If so, describe these changes and explain why you made them.
    - How have these strategies changed as he or she aged?

Citations: Holly, Porter, Kamienski, & Lim, 2019; Monuteaux, Azrael, & Miller, 2019; Ngo et al., 2019; Wallace, 2019
Research Question: What are Appalachian caregivers' gun storage practices?

5. Does your child express interest in the guns in your home?

- If so, describe this interest.
- If not, why do you think that?
- Describe how your child was introduced to firearms.

Citations: Ablewhite et al., 2015; Baxley & Miller, 2006; Webster, Wilson, Duggan, & Pakula, 1992
Research Question: What are Appalachian caregivers' perceptions of child interest in firearms in the home?

6. Describe any gun safety conversations you or another adult has had with your child.
    - Who talks with your child about gun safety?
    - When did these conversations start?
    - What are some of the safety topics discussed?
    - What are some of the questions your child has asked about gun safety?
    - In your opinion, does having a gun in the house make these conversations more or less important?
        - Why or why not?
    - Ideally, what is the best way to teach children about gun safety in the home?
    - Ideally, who should teach children about gun safety in the home?

Citations: Holly et al., 2019; Wallace, 2019
Research Question: What are Appalachian caregivers' experiences teaching gun safety to children?

7. Ideally, how should caregivers address child safety when there are guns in the home?
    - Describe different strategies they should use.
    - Do these strategies change as children age?
        - Why or why not?
    - Where should caregivers get reliable information on gun safety?

- What is the most important thing that a caregiver can do to keep their child safe around guns in the home?

Research Question: What are Appalachian caregivers' attitudes toward gun safety in the home?

8. Conclusion
   - Thank you for taking the time to tell me your experiences and perspectives.
   - As I mentioned at the beginning of this interview, your answers will be kept confidential.
   - Thank you again. Here is a gift card as a thank you for your time and willingness to share your experiences.

References

1. Ablewhite J, Peel I, McDaid L, Hawkins A, Goodenough T, Deave T, Stewart J, Kendrick D. Parental perceptions of barriers and facilitators to preventing child unintentional injuries within the home: A qualitative study. BMC Public Health 2015;15(1):280. https://doi.org/10.1186/s12889-015-1547-2

2. Baxley F, Miller M. Parental misperceptions about children and firearms. Archives of Pediatrics & Adolescent Medicine 2006;160(5):542-7. https://doi.org/10.1001/archpedi.160.5.542

3. Holly C, Porter S, Kamienski M, Lim A. School-based and community-based gun safety educational strategies for injury prevention. Health promotion practice 2019;20(1):38-47. https://doi.org/10.1177/1524839918774571

4. Monuteaux MC, Azrael D, Miller M. Association of increased safe household firearm storage with firearm suicide and unintentional death among US youths. JAMA Pediatrics 2019;173(7):657-62. https://doi.org/10.1001/jamapediatrics.2019.1078

5. Ngo QM, Sigel E, Moon A, Stein SF, Massey LS, Rivara F, King C, Ilgen M, Cunningham R, Walton MA, FACTS Consortium. State of the science: a scoping review of primary prevention of firearm injuries among children and adolescents. Journal of Behavioral Medicine 2019;42(4):811-29. https://doi.org/10.1007/s10865-019-00043-2

6. Wallace LN. Gun safety discussions with caregivers: Timing and demographic associations in a retrospective study. BMJ Evidence-Based Medicine 2020;25(2):1-2. https://doi.org/10.1136/bmjebm-2019-111223

7. Webster DW, Wilson ME, Duggan AK, Pakula LC. Parents' beliefs about preventing gun injuries to children. Pediatrics 1992;89(5):908-14.
